# Long Noncoding RNA DANCR Activates Wnt/β-Catenin Signaling through MiR-216a Inhibition in Non-Small Cell Lung Cancer

**DOI:** 10.3390/biom10121646

**Published:** 2020-12-08

**Authors:** Justine E. Yu, Julia A. Ju, Nicholas Musacchio, Trevor J. Mathias, Michele I. Vitolo

**Affiliations:** 1 Program in Molecular Medicine, University of Maryland Graduate Program in Life Sciences, Baltimore, MD 21201, USA; trevor.mathias@som.umaryland.edu; 2 Marlene and Stewart Greenebaum NCI Comprehensive Cancer Center, University of Maryland School of Medicine, Baltimore, MD 21201, USA; jju@som.umaryland.edu; 3 Department of Biochemistry and Molecular Biology, University of Maryland School of Medicine, Baltimore, MD 21201, USA; nicholas.musacchio@som.umaryland.edu; 4 Department of Pharmacology, University of Maryland School of Medicine, Baltimore, MD 21201, USA

**Keywords:** Wnt, β-catenin, lncRNA, microRNA, non-small cell lung cancer

## Abstract

Long noncoding RNA differentiation antagonizing nonprotein coding RNA (lncRNA-DANCR) is associated with poor prognosis in multiple cancers, and promotes cancer stemness and invasion. However, the exact mechanisms by which DANCR promotes non-small cell lung cancer (NSCLC) remain elusive. In this study, we determined that DANCR knockdown (KD) impeded cell migration and reduced stem-like characteristics in two NSCLC cell lines, A549 and H1755. Wnt signaling was shown to promote NSCLC proliferation, stemness, and invasion; therefore, we hypothesized that DANCR may regulate these activities through induction of the Wnt/β-catenin pathway. DANCR KD reduced β-catenin signaling and protein expression, and decreased the expression of β-catenin gene targets c-Myc and Axin2. One of the well-defined functions of lncRNAs is their ability to bind and inhibit microRNAs. Through in silico analysis, we identified tumor suppressor miR-216a as a potential binding partner to DANCR, and confirmed this binding through coimmunoprecipitation and luciferase-reporter assays. Furthermore, we show that DANCR-induced β-catenin protein expression may be blocked with miR-216a overexpression. Our findings illustrate a role of DANCR in NSCLC migration and stemness, and suggest a novel DANCR/miR-216a signaling axis in the Wnt/β-catenin pathway.

## 1. Introduction

Lung cancer is the leading global cause of cancer-related deaths. Non-small cell lung cancer (NSCLC) comprises 85% of all lung cancer cases [1]. Despite advancements in therapy for early-stage NSCLC, the five-year relative survival rate of NSCLC remains at 23% because the majority of patients are diagnosed at advanced stages, when cancer cells have already metastasized [1]. Currently, there are limited therapies for metastatic NSCLC. Therefore, further investigation on the mechanisms of NSCLC progression is necessary to improve patient outcomes.

Noncoding RNAs (ncRNAs) are critical regulators of gene expression that are commonly dysregulated in cancer. MicroRNAs (miRNAs) are small ncRNAs that are 16–24 nucleotides long, and function post-transcriptionally by binding to target mRNAs at specific recognition sequences, leading to target mRNA degradation or translational repression. Several miRNAs have been shown to play important regulatory roles through the promotion or suppression of NSCLC [2]. In particular, miR-216a was described as an inhibitor of NSCLC cell growth, invasion, and metastasis [3]. However, the direct mechanism by which miR-216a expression is regulated in NSCLC has not been defined.

Long noncoding RNAs (lncRNAs) are a heterogeneous group of regulatory ncRNAs defined by their lack of protein-coding potential and minimal length of 200 nucleotides. LncRNAs differ from miRNAs by their ability to bind to a wide variety of macromolecules (i.e., protein, RNA, and DNA) to exert diverse functions. LncRNA differentiation-antagonizing nonprotein-coding RNA (DANCR) was first identified as a suppressor of epidermal progenitor differentiation [4]. In the last few years, multiple studies established its role in promoting cancer stem cell (CSC) function, tumor growth, survival, and migration in several cancers [5,6,7,8,9,10]. In NSCLC, DANCR was shown to promote tumorigenesis by inhibiting several tumor suppressor miRNAs [11,12,13]. However, the mechanisms by which DANCR promotes NSCLC tumorigenesis and progression are unknown.

Wnt signaling is a key pathway that normally functions in development and stemness [14]. Aberrations in Wnt signaling have been connected to unregulated proliferation, cancer development, stem cell maintenance, and metastasis in multiple cancer types. In the inactive canonical Wnt pathway, when Wnt signaling is inactive, β-catenin is held in the cytoplasm in a destruction complex composed of scaffolding proteins Axis inhibition protein (Axin), adenomatous polyposis coli (APC), and disheveled (Dvl), and kinases glycogen kinase 3-beta (GSK-3β) and casein kinase-alpha (CK1α) that phosphorylate β-catenin, leading to its proteolytic degradation. Wnt signaling is activated when a Wnt ligand binds to a Frizzled (Fzd) receptor, which forms a complex with coreceptor lipoprotein receptor-related protein (LRP). The receptor complex recruits Axin and Dvl, disrupting the destruction complex. This allows for β-catenin to accumulate in the cytoplasm and translocate to the nucleus, where it binds to T-cell factor (TCF)/lymphoid enhancer-binding factor (LEF) transcription factors and activates the transcription of Wnt target genes such as c-myc and Axin2. Given the important role of Wnt in lung homeostasis, it is not surprising that alterations in Wnt/β-catenin signaling have also been shown to majorly impact NSCLC tumorigenesis and progression [15].

In this study, we report that DANCR knockdown (KD) reduces migration, cell viability, and stem-like characteristics. We also show that DANCR overexpression in NSCLC activates Wnt/β-catenin signaling, which can be effectively blocked by miR-216a overexpression. Furthermore, we confirm the interaction between DANCR and miR-216a. For the first time, we show that Wnt/β-catenin activation can be mediated through the DANCR repression of miR-216a, thereby establishing a novel DANCR/miR-216a/Wnt signaling axis that promotes migration and stem cell characteristics that are fundamental to NSCLC progression.

## 2. Materials and Methods

### 2.1. Cell Culture

NSCLC adenocarcinoma cell lines A549, H1975, H1755, H1944, H2087, and H358, and NSCLC large cell carcinoma cell lines H661 and H1299 were maintained in RPMI 1640 + l-glutamine (Corning, Sigma-Aldrich, St. Louis, MO, USA) supplemented with 10% fetal bovine serum (FBS, Atlanta Biologicals), 5 mg/mL D-glucose (Sigma-Aldrich, St. Louis, MO, USA), 5 mM HEPES (Invitrogen, ThermoFisher Scientific, Waltham, MA, USA), and 0.05 mM sodium pyruvate (Sigma). HBE2 was maintained in Keratinocyte SFM without calcium chloride medium (Gibco, ThermoFisher Scientific, Waltham, MA, USA). Cells were maintained in a humidified environment at 37 °C and 5% CO_2_. Cell lines A549, H1755, H1944, and H2087 were obtained from ATCC (Manassas, VA, USA). H1975, H661, H1299, H358, and HBE2 were a generous gift from Dr. Feng Jiang, University of Maryland School of Medicine. All used cell lines regularly tested negative for mycoplasma contamination throughout the whole duration of this study.

### 2.2. The Cancer Genome Atlas (TCGA) Analysis

The publicly available The Cancer Genome Atlas (TCGA) dataset was evaluated using the UCSC Xena Functional Genomics Browser. Analyzed datasets were TCGA lung adenocarcinoma (*n* = 706) and lung squamous cell carcinoma (*n* = 626) by RNAseq (Illumina HiSeq, Illumina, Inc., San Diego, CA, USA).

### 2.3. qPCR

Total RNA was extracted with TRIzol reagent (Invitrogen). mRNA was converted to cDNA using M-MLV reverse transcriptase (Invitrogen) and random hexamer primers. MiRNAs were converted to cDNA using miScript II RT kit (Qiagen, Redwood City, CA, USA). qRT-PCR for miR-216a was normalized to RNU6 using miScript Primer assays and miScript Sybr Green PCR Kit (Qiagen). qRT-PCR for gene expression was normalized to GAPDH expression, and performed using Light Cycler Sybr Green I Master kit (Roche, Basel, Switzerland). Gene primers used in the study were:

- *DANCR:* F: 5′-CGTCTCTTACGTCTGCGGAA-3′
     R: 5′-GGACACGTGGTTGCTACAAG-3′
- *SOX2:* F: 5′-GCTTTTGTTCGATCCCAACTTTC-3′
     R: 5′-ATGGATTCTCGGCAGACTGATTC-3′
- *ALDH1:* F: 5′-ATGTCTGGAAATGGAAGAGAACTGG-3′
     R: 5′-GTGACTGTAAGGAGATGCTTAGCTATTGAA-3′
- *CMYC:* F: 5′-GCTGCTTAGACGCTGGATTT-3′
     R: 5′-CACCGAGTCGTAGTCGAGGT-3′
- *AXIN2:* F: 5′-CAAGGGCCAGGTCACCAA-3′
     R: 5′-CCCCCAACCCATCTTCGT-3′
- *GAPDH:* F: 5′-AAGGTGAAGGTCGGAGTCAA-3′
     R: 5′-AATGAAGGGGTCATTGATGG-3′

### 2.4. SiRNA Transfection

Cells were seeded at 3 × 10^5^ cells/well in a 6 well plate in 2 mL of medium. SiRNA transfection was performed with 40 nM of 5′-Cy5-tagged or untagged siRNAs using Lipofectamine RNAiMAX (ThermoFisher Scientific) according to the manufacturer’s instructions, and samples were harvested 48–72 h post-transfection. SiRNAs were obtained from Sigma-Aldrich. SiRNA sequences used in the study were:

- *Scramble siRNA:* 5′-UAA CUC GCU CGA AGG AAU C-3′
- *DANCR siRNA-1:* 5′-GCG UAC UAA CUU GUA GCA A-3′
- *DANCR siRNA-2*: 5′-UAA CAG AAU CCA CCU CCG A-3′

### 2.5. Establishment of Stable lncRNA-DANCR Knockdown Cell Lines

Scramble control shRNA and the pLV-hU6-EF1α-puro backbone were purchased from Biosettia, and DANCR shRNA constructs were produced using the following target sequences: 5′-GCCGGTCATGAGATTATAT-3′ for sh278 and 5′-AAAAGGCCAAATATGCGTACTAA-3′ for sh321. Stable infections were performed for lentiviral constructs: plv-hu6-EF1a-puro-negative control shRNA, plv-hu6-DANCRsh278, and plv-hu6-EF1a-puro-DANCRsh321. Briefly, HEK293T cells were transfected using the Lenti-X Packaging Single Shots (Takara Bio USA, Inc., Mountain View, CA, USA) following the manufacturer’s protocol. After 24 h, the medium was changed and lentivirus-containing supernatants were harvested at 48 and 72 h post transfection. A549 cells were infected with the virus at an MOI of 10 with 4 μg/mL polybrene, and pooled clones were then selected with puromycin (1 μg/mL).

### 2.6. Two-Dimensional Colony Formation

A549 (10^3^) and H1755 (10^4^) cells were seeded in 100 mm plates and grown at 37 °C and 5% CO_2_. After 2 weeks, colonies were fixed and stained in 10% formalin with 0.05% Crystal Violet, and then quantified using automated colony counter ProtoCOL3.

### 2.7. Cell Viability

For cell-viability assays, cells were trypsinized and counted 24 h after siRNA transfection. A549 (500 cells/well) and H1755 (2500 cells/well) were seeded in triplicate in 96 well plates, and viability was measured using the CellTiter 96^®^ AQueous One Solution Cell Proliferation Assay (Promega, Madison, WI, USA) as per manufacturer instructions. Cells were incubated with the CellTiter reagent for 1 h and then read at 490 nm every day for 5 days.

### 2.8. Cell Impedance Assay

Cells were trypsinized and counted 24 h after siRNA transfection. A549 or H1755 cells (4 × 10^4^) were plated in triplicate into the RTCA xCELLigence microwell CIM plates (ACEA Biosciences), and placed in the RTCA DP Analyzer located in a humidified incubator maintained at 37 °C and 5% CO_2_. Filters in the CIM plate chambers contained electrodes that could measure the change in impedance as cells passed through the filter and adhered to its underside. The change in impedance (shown as cell index) is a direct measure of number of cells that migrated. Readings were taken in real time every 15 min for 72 h.

### 2.9. Anchorage-Independent Tumorsphere Growth

Cells were trypsinized and passed through a 40 µm cell strainer to obtain a single cell suspension. Cells were then seeded in 6-well plates coated with 2% polyHEMA (Sigma) at a dilution of 1000 cells/mL in 3 mL of RPMI media containing 2% B27, 20 ng/mL EGF, 4 μg/mL insulin, and 0.4% BSA. Spheres larger than 50 μm were counted after 7 days of culture.

### 2.10. Western Blotting

Total cell lysates (30 μg) were separated by SDS-PAGE and transferred onto a polyvinylidene difluoride (PVDF) membrane. The membrane was incubated with a specific primary antibody using manufacturer’s recommended dilutions overnight followed by the horseradish peroxidase (HRP)-conjugated secondary antibody (1:5000) and visualized by the ECL Western blotting detection system (Thermo Scientific). The following antibodies were used from Santa Cruz Biotchnology, Inc. (Dallas, TX, USA): Sox2 (sc-365823), β-catenin (sc-7963), GAPDH (sc-32233); Cell Signaling: c-Myc (CST-5605), Axin2 (CST-5863); and Sigma: β-actin (A5441).

### 2.11. Luciferase-Reporter Assay

For the TOPFlash luciferase reporter assay, 1 × 10^5^ A549 cells/well were plated the day before in a 12-well plate, and then cotransfected with 8X Super TOPFlash plasmid (Addgene 12456), Renilla vector, and either with Control shRNA, DANCR sh278, or DANCR sh321 vectors using a FuGene HD (Promega) reagent according to manufacturer’s instructions. For the lncRNA-DANCR luciferase reporter construct, DANCR cDNA was amplified using the following primers: 5′-ACTGATGCTAGCGTTGACAACTACAGGCACAAG-3′ and 5′-ATCAGTCTCGAGGTCAG GCCAAGTAAGTTTATTAAC-3′, and cloned into the psGG 3′UTR promoter downstream of luciferase using NheI and XhoI sites; the inserted sequence was verified by sequencing to the truncated WT lncRNA-DANCR (nt271-nt915) containing the miR-216a MRE site 5′-TGAGATTA-3′ at nt774-nt781. The day before, 293T cells were plated in a 12 well plate and then cotransfected with pSGG-luc-DANCR construct and the Renilla vector, along with the p-Babe-miR-216a plasmid (Addgene 65054) using Lipofectamine 2000. Luciferase-reporter assays were performed 48 h after transfection using the dual luciferase assay system (Promega, Madison, WI, USA) according to manufacturer’s instructions, and normalized to Renilla luciferase activity.

### 2.12. Prediction of RNA Interactions

DIANA LncBase Predicted v.2 and miRanda-mirSVR tools were used to identify potential miRNAs that interacted with DANCR. TargetScan v7.2 was used to predict potential miR-216a mRNA targets.

### 2.13. MS2–MCP Immunoprecipitation

Stable A549 DANCR KD cells (3 × 10^5^ cells/well of a 6-well plate) were cotransfected with phage-ubc-nls-ha-tdMCP-GFP (Addgene 40649) and either pcDNA3-MS2-DANCR or pcDNA3-MS2-Vector constructs using FuGeneHD according to manufacturer’s instructions. After 48 h of transfection, cells were lysed and incubated while rocking with anti-GFP conjugated beads for 2 h at room temperature. Pulldown products were released using Trizol, and RNA was extracted and used to measure miR-216a expression via qRT-PCR.

### 2.14. Dual DANCR and MiR-216a Overexpression

The DANCR sequence was amplified by PCR using the following primers: 5′-ACTGATGGATCCCCCGCCCCGCGC-3′ and 5′-ACTGATGAATTCGTCAGGCCAAGTAAG TTTATTAACCTGCC-3′, and then cloned into the pBABE-puro vector using the BamHI and EcoRI sites. DANCR overexpression was achieved by transfecting A549 cells (3 × 10^5^ cells/well of a 6-well plate) with pBABE-puro vector or pBABE-puro-DANCR plasmids using FuGENE HD reagent according to the manufacturer’s instructions. Inserted DANCR was confirmed by sequencing to be WT DANCR (nt61-nt915) containing miR-216a MRE site 5′-TGAGATTA-3′ at nt774-nt781. Cells were incubated for 48 h, expanded, and plated for second consecutive transfection of pBABE-puro vector or pBABE-puro-miR-216a. Lysates and RNA were harvested 48 h after the second transfection.

## 3. Results

### 3.1. LncRNA-DANCR Is Highly Upregulated in NSCLC Tumors and Cell Lines

**Figure 1:**
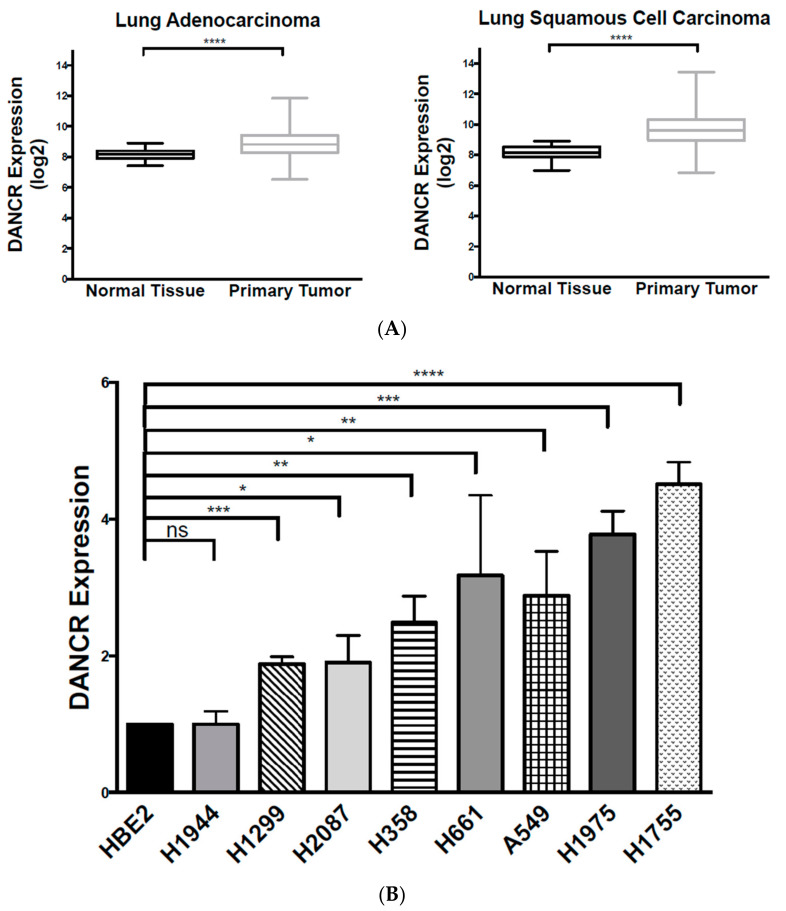
LncRNA-DANCR is highly upregulated in NSCLC tumors and cell lines. (**A**) Analysis of The Cancer Genome Atlas data for DANCR expression in primary lung adenocarcinoma (*n* = 706) and squamous cell carcinoma (*n* = 626) compared to normal lung tissue (*t* test with Welch’s correction, **** *p* < 0.0001); (**B**) qRT-PCR measuring fold change of DANCR expression in NSCLC cell lines compared to lung epithelial cell line HBE2 normalized to 1 (*n* = 3, *t* test, * *p* < 0.05, ** *p* < 0.01, *** *p* < 0.001, **** *p* < 0.0001).

Despite advancements in therapy for early-stage NSCLC, prognosis for patients with advanced-stage disease remains poor due to limited therapeutic options. Therefore, there is an inherent need to identify molecular targets for more effective therapies. In order to illuminate the molecular mechanisms that promote NSCLC progression, we examined The Cancer Genome Atlas (TCGA) dataset for DANCR expression in the most common NSCLC subtypes: lung adenocarcinoma (*n* = 706) and lung squamous cell carcinoma (*n* = 626). This analysis illustrated that DANCR expression was highly upregulated in lung adenocarcinoma and squamous cell carcinoma compared to normal lung tissue (Figure 1A). We further examined DANCR expression in NSCLC adenocarcinoma cell lines A549, H1975, H1755, H1944, H2087, and H358, and NSCLC large cell carcinoma cell lines H661 and H1299, and found that DANCR was significantly increased in 7 of the 8 NSCLC cell lines compared to human bronchial epithelial cell line HBE2 (Figure 1B). Together, these data demonstrate that lncRNA-DANCR is highly upregulated in NSCLC.

### 3.2. Knockdown of LncRNA-DANCR Expression in NSCLC Cells Inhibits Long-Term Growth and Migration

**Figure 2:**
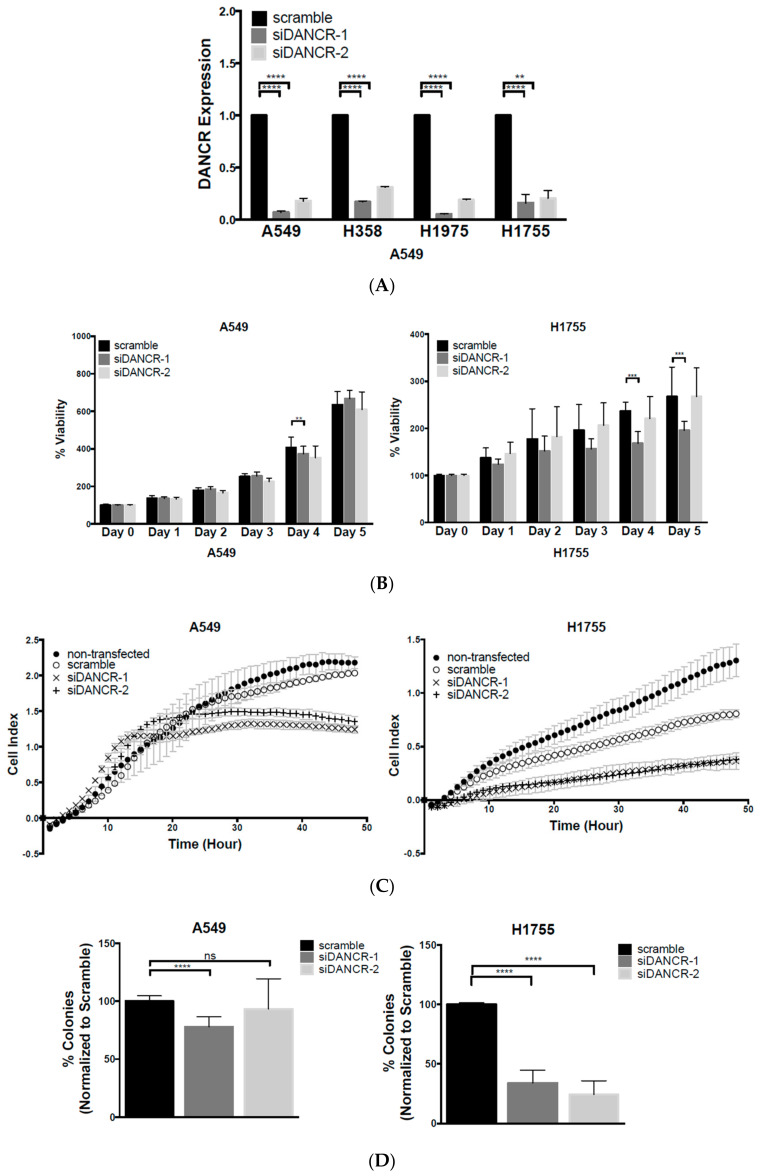
Knockdown (KD) of lncRNA-DANCR expression in NSCLC cells inhibits migration and clonogenic growth. (**A**) Fold change of DANCR expression in NSCLC cell lines A549, H358, H1975, and H1755 transiently transfected with two different DANCR siRNAs (*n* = 3, *t* test, ** *p* < 0.01, **** *p* < 0.0001); (**B**) effect of DANCR KD on cell growth of A549 and H1755 cells using Aqueous Cell Titer 96 reagent (*n* = 3, 2-way ANOVA, ** *p* < 0.01, *** *p* < 0.001); (**C**) cell impedance assay using xCELLigence RTCA SP system on A549 and H1755 DANCR KD cells compared to scramble control (*n* = 3); (**D**) colony-formation assay on A549 and H1755 two weeks after seeding (*n* = 3, *t* test, **** *p* < 0.0001).

In order to investigate the functional role of DANCR in NSCLC, we employed transient methods to knockdown (KD) DANCR expression. Two distinct DANCR siRNAs were transfected into NSCLC adenocarcinoma cells A549, H1975, H358, and H175,5 yielding efficient KD of DANCR expression (Figure 2A). We selected two cell lines, A549 and H1755, for further experimentation.

We first examined the effect of DANCR KD on cell proliferation. A549 and H1755 cells were plated 24 h post-siRNA transfection, and monitored for daily growth for 5 days at 24 h intervals using Aqueous Cell Titer 96 assay (Figure 2B). For every experiment, a portion of transfected cells were harvested for qRT-PCR to verify DANCR KD. Cells with DANCR KD resulting from siDANCR-1 showed minimal decrease in proliferation on Days 4 (A549 and H1755 cells) and 5 (H1755 cells).

We then used cell impedance assays to examine the effect of DANCR inhibition on cell motility. The xCELLigence Impedance assay displays the migration of cells in real time by providing an increased change in impedance as cells move through the filter from the top to the bottom chamber. DANCR KD reduced the migration of both A549 and H1755 cells (Figure 2C). Since transient DANCR KD had only a small and delayed effect on proliferation, the effects on migration were not due to any differences in proliferation over the course of the migration experiment. This indicated that the effects of DANCR KD on migration are independent of cell growth. Additionally, we created stably expressing A549 DANCR KD cells using lentiviral transduction of two different DANCR-shRNAs (Figure S1A). We determined that stable DANCR KD also inhibited migration, which was still independent of growth inhibition in A549 cells (Figure S1B,C).

We next assessed the effect of DANCR KD on NSCLC long-term clonogenic potential using two-dimensional colony formation assays. Transient reduction of DANCR expression with siRNAs in the A549 cells resulted in a minor but significant inhibition of colony formation, but again with only siDANCR-1 (Figure 2D and Figure S2A). The examination of stable DANCR KD clones did not show any differences in colony formation compared to the scrambled controls (Figure S1D), leading to the conclusion that growth inhibition may not be specific to reduced DANCR expression in A549 cells. However, the transient reduction of DANCR expression strongly inhibited colony formation of H1755 cells (Figure 2D and Figure S2A). This suggests that reduced expression of DANCR can induce short-term changes in proliferation and long-term effects on cell growth that may be cell line-dependent.

### 3.3. Knockdown of LncRNA-DANCR Expression in NSCLC Cells Inhibits Stem-Cell Characteristics

**Figure 3:**
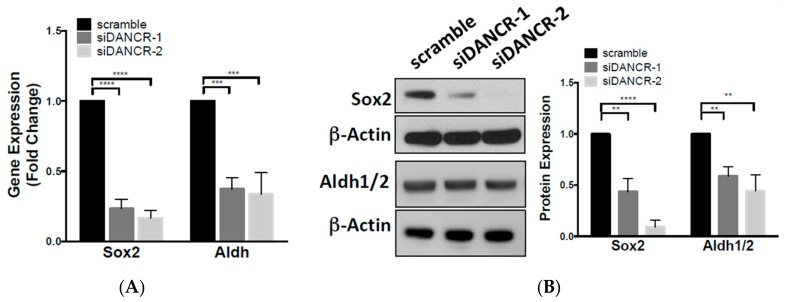

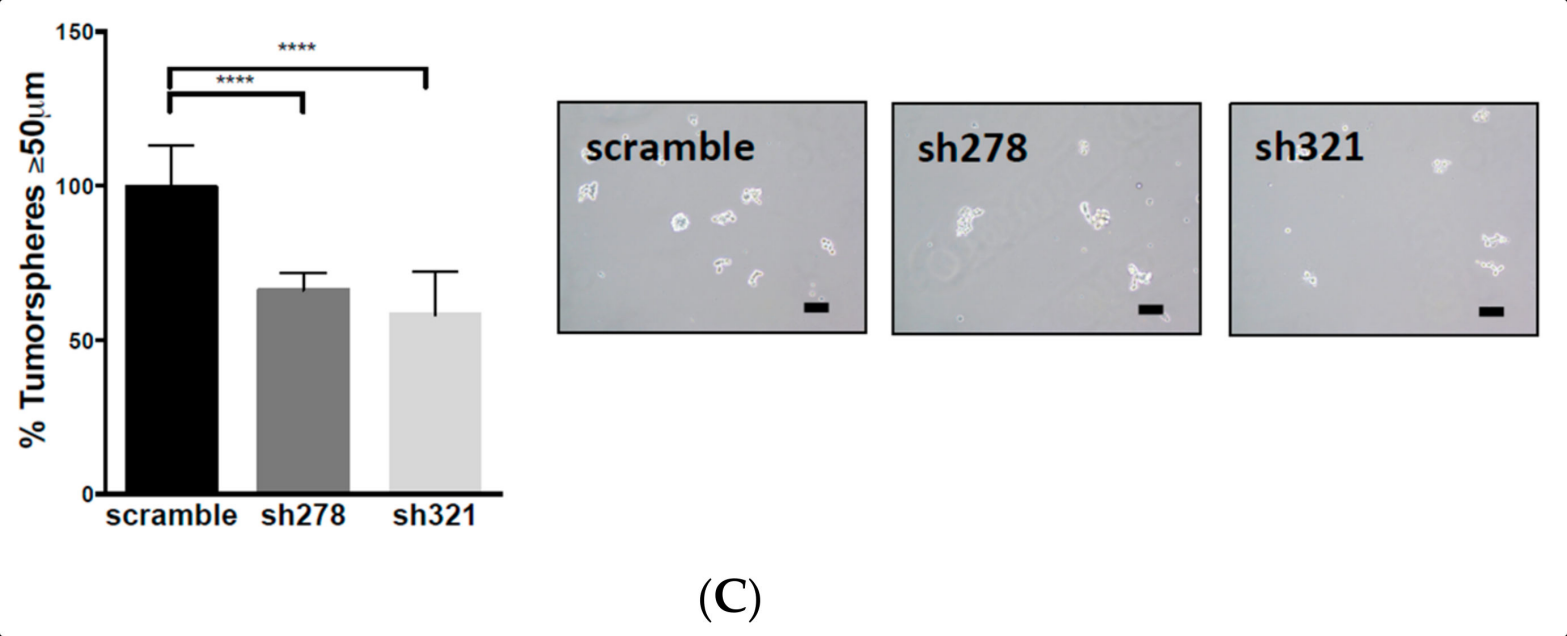
Knockdown of lncRNA-DANCR expression in NSCLC cells inhibits stem-cell characteristics. (**A**) Fold change of *SOX2* and *ALDH* gene expression in A549 DANCR KD cells compared to scramble control (*n* = 3, *t* test, *** *p* < 0.001), *n* = 3; *t* test, **** *p* < 0.0001); (**B**) fold change of Sox2 and Aldh1/2 protein expression in A549 DANCR KD cells compared to scramble control (*n* = 3; *t* test, ** *p* < 0.01, **** *p* < 0.0001); (**C**) tumorsphere formation in stable A549 DANCR KD cells compared to scramble control. Representative images of tumorspheres shown, scale bar = 100 γm (*n* = 3, *t* test, **** *p* < 0.0001).

Cells that exhibit migratory capacity possess phenotypic similarities with the cancer stem cell (CSC) subpopulation. Stem-like programs are regulated by the timing and expression patterns of multiple markers. To investigate the effect of DANCR KD on stem-like characteristics, we evaluated the expression of two fundamental CSC markers, SOX2 and ALDH1/2. We found that transient DANCR KD significantly reduced SOX2 and ALDH mRNA, and protein expression compared to scramble control (Figure 3A,B).

Given the significant effect of DANCR KD on reducing Sox2 expression, and the essential role of Sox2 in stem cell self-renewal and pluripotency [16], we next assessed possible differences in the CSC population due to DANCR. Using stable A549 DANCR KD cells (Figure S1A), we assessed the ability of A549 cells to form tumorspheres in serum-free anchorage-independent conditions. Stable KD of DANCR expression in A549 cells reduced the percentage of tumorspheres ≥ 50 µm that formed without affecting tumorsphere size (Figure 3C). These results indicated that DANCR contributes to NSCLC stem cell self-renewal.

### 3.4. LncRNA-DANCR Regulates Wnt/β-Catenin Signaling

**Figure 4:**
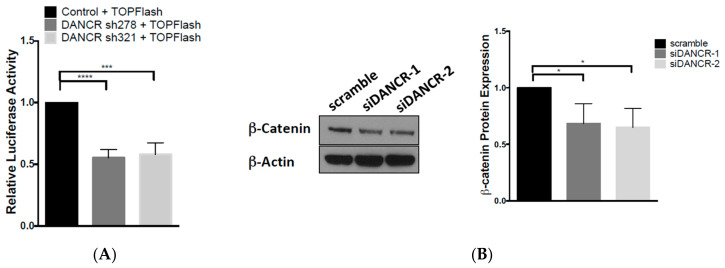

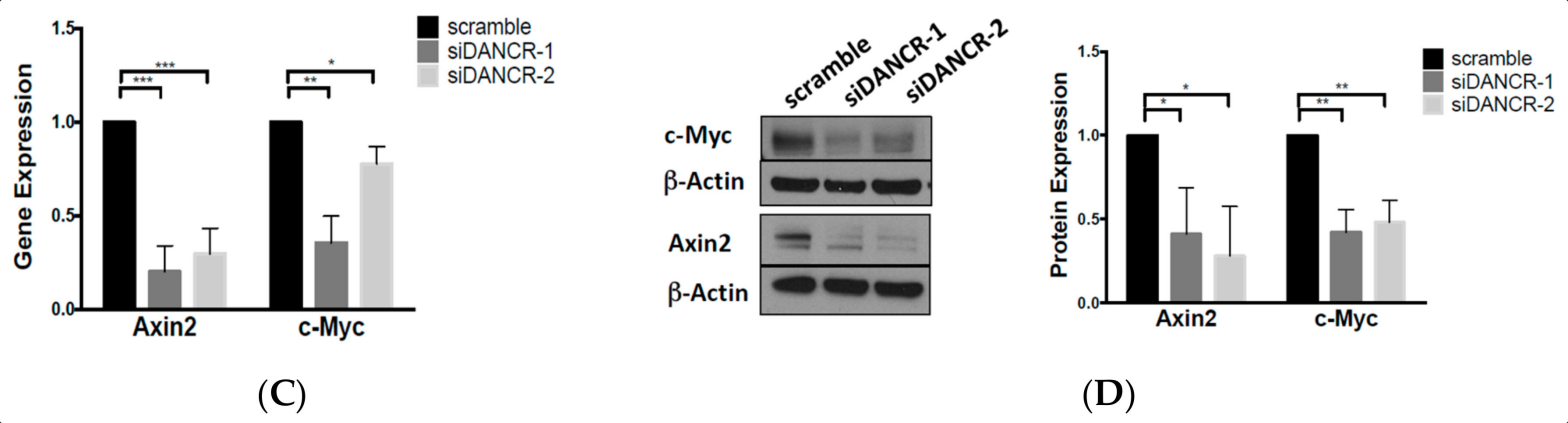
LncRNA-DANCR regulates Wnt/β-catenin signaling. (**A**) β-Catenin activity in A549 cells cotransfected with TOPFlash luciferase reporter + DANCR shRNAs compared to cells transfected with TOPFlash luciferase reporter + scramble control (*n* = 3, *t* test, *** *p* < 0.001, **** *p* < 0.0001); (**B**) fold change of β-catenin protein expression in A549 DANCR KD cells compared to scramble control (*n* = 3, *t* test, * *p* < 0.05); (**C**) fold change of *CMYC* and *AXIN2* gene expression in A549 DANCR KD cells compared to scramble control (*n* = 3, *t* test, * *p* < 0.05, ** *p* < 0.01, *** *p* < 0.001); (**D**) fold change of c-myc and Axin2 protein expression in A549 DANCR KD cells compared to scramble control (*n* = 3, *t* test, * *p* < 0.05, ** *p* < 0.01).

The canonical Wnt/β-catenin pathway was shown to promote cancer cell proliferation, invasion, and stemness in many types of cancers including colorectal, breast, and leukemia [17]. We analyzed the impact of DANCR on β-catenin signaling activation with a luciferase-reporter assay using the β-catenin reporter Super 8X-TOPFlash plasmid, which contains TCF/LEF sites upstream of a luciferase reporter. Transient knockdown of DANCR expression in A549 cells with shRNAs reduced luciferase activity by approximately 50% compared to the control shRNA (Figure 4A and Figure S1E). We then assessed changes in Wnt signaling molecules, and found that the transient KD of DANCR reduced β-catenin protein expression (Figure 4B). We further explored the expression of downstream Wnt target genes Axin2 and c-Myc, and found that both mRNA and protein expression were downregulated upon DANCR KD (Figure 4C,D). Together, these results indicated that DANCR regulates Wnt/β-catenin signaling.

### 3.5. LncRNA-DANCR Is a Competitive Inhibitor of MiR-216a

**Figure 5:**
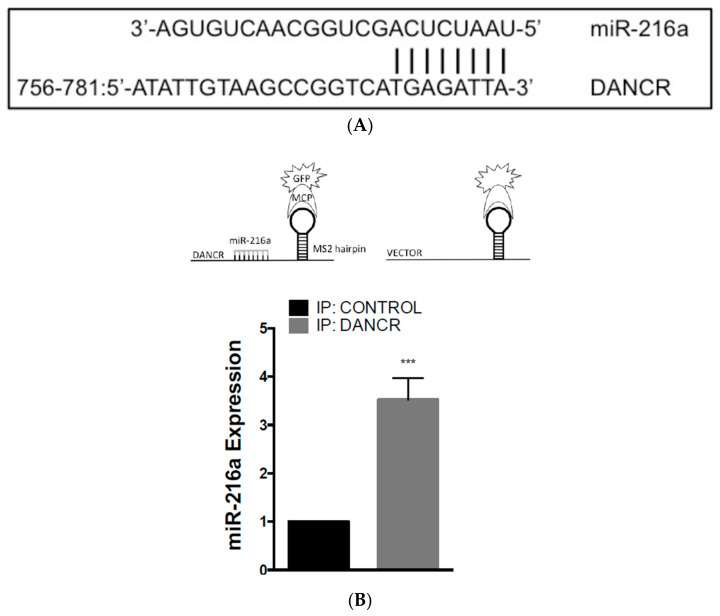

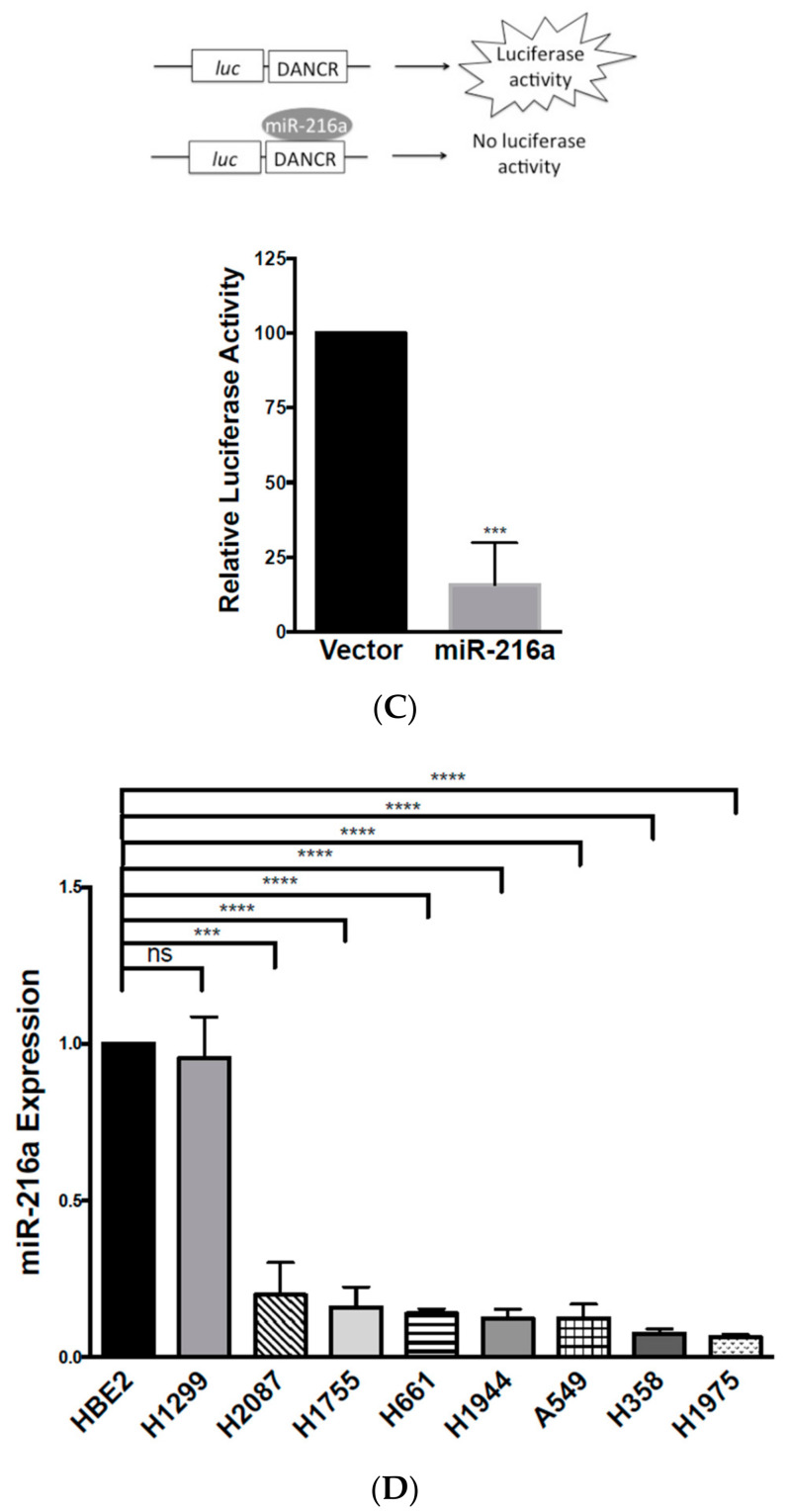
LncRNA-DANCR is a competitive inhibitor of miR-216a. (**A**) Potential binding position of DANCR MRE and miR-216a; (**B**) (top) schematic of MCP-MS2 immunoprecipitation assay; (bottom) MCP–MS2 immunoprecipitation assay using A549 DANCR KD cells transfected with MS2-DANCR compared to MS2-control cells (*n* = 3, *t* test, *** *p* < 0.001); (**C**) (top) schematic of luciferase-DANCR reporter assay; (bottom) 293T cells cotransfected with luciferase-DANCR reporter + miR-216a expression plasmid compared to luciferase-DANCR reporter + vector control cells (*n* = 3, *t* test, *** *p* < 0.001); (**D**) fold change of miR-216a expression in NSCLC cell lines compared to lung epithelial cell line HBE2 (*n* = 3, *t* test, *** *p* < 0.001, **** *p* < 0.0001).

LncRNAs have multiple properties, which include their ability to act as competitive endogenous RNA (ceRNA). CeRNAs are able to inhibit miRNA activity by competitively binding miRNAs through miRNA recognition elements (MRE), resulting in inhibition of miRNA–mRNA binding and the upregulation of the miRNA targets. Using in silico analysis, DANCR was predicted to contain an MRE site for miR-216a (Figure 5A). Previous reports showed that miR-216a reduced migration and invasion in glioma, and colorectal and pancreatic cancer [11,18,19]. Additionally, Wang, RT et al. reported that miR-216a was downregulated in NSCLC, which promoted NSCLC growth, invasion, and metastasis in mice [11].

To further evaluate DANCR and miR-216a interaction, we employed the MS2–MCP (MS2-MS2 Bacteriophage Coat Protein) system, in which GFP-tagged MCP binds to MS2 stem-loop sequences that can consequently be used in immunoprecipitation studies to assess RNA binding. Stable A549 DANCR KD cells were cotransfected with an MCP-GFP tagged plasmid with either an MS2-DANCR or MS2-Vector plasmid. Then, GFP was immunoprecipitated, and RNA was extracted to evaluate miR-216a pulldown and association. These results demonstrated that miR-216a was pulled down almost fourfold more in MS2-DANCR cells than in MS2-Vector cells (Figure 5B). Additionally, a luciferase-reporter assay was used to assess the binding of miR-216a to the DANCR MRE site. HEK-293T cells were transfected with a luciferase-reporter plasmid that contained the DANCR MRE site upstream of the luciferase reporter. Cells overexpressing miR-216a had an over 80% reduction in relative luciferase activity compared to that in vector control cells (Figure 5C).

Moreover, we hypothesized that the upregulation of DANCR in NSCLC has reciprocal downregulation of miR-216a expression. As expected, miR-216a was downregulated in a number of different NSCLC cell lines, including H2087, H1755, H661, H1944, A549, H358, and H1975 compared to HBE2 cells (Figure 5D). Taken together, these results provide evidence that DANCR competitively binds miR-216a, and DANCR may regulate miR-216a expression.

### 3.6. DANCR Promotes Wnt/β-Catenin Signaling through MiR-216a Inhibition

**Figure 6:**
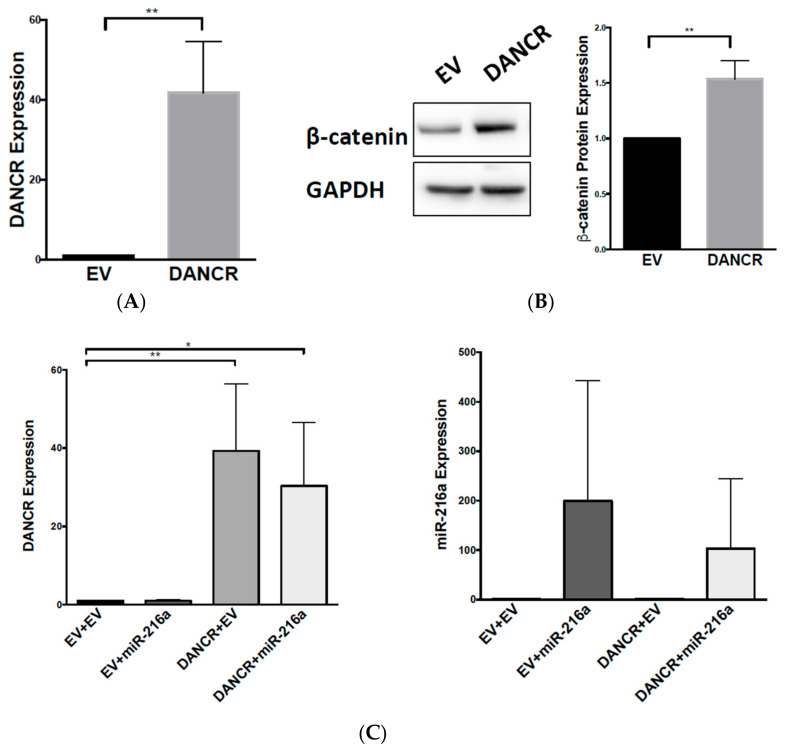

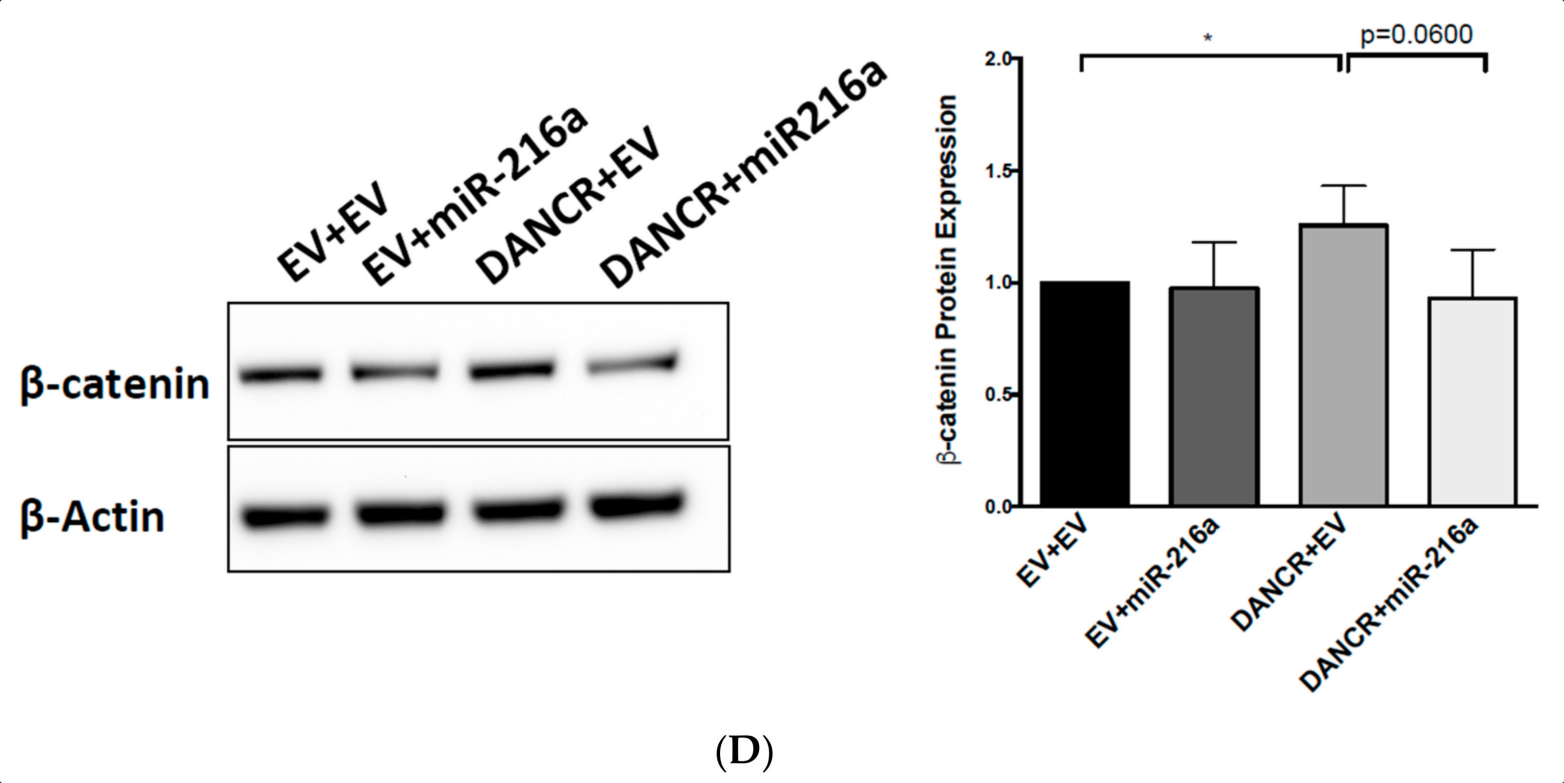
DANCR promotes Wnt/β-catenin signaling through miR-216a inhibition. (**A**) qRT-PCR measuring fold change of DANCR expression in A549 cells transfected with pBABE-DANCR compared to vector control (*n* = 3; *t* test, ** *p* < 0.001); (**B**) fold change of β-catenin protein expression in A549 DANCR overexpressing cells (*n* = 3, *t* test, ** *p* < 0.001); (**C**) (left) qRT-PCR measuring fold change of DANCR expression in A549 cells doubly transfected with vector + vector (EV + EV), vector + pBABE-miR-216a (EV + miR-216a), vector + pBABE-DANCR (EV + DANCR), or pBABE-DANCR + pBABE-miR-216a (DANCR + miR-216a); (right) qRT-PCR measuring fold change of miR-216a expression (*n* = 4, *t* test, * *p* < 0.05, ** *p* < 0.001); (**D**) fold change of β-catenin protein expression in A549 cells doubly transfected with vector + vector (EV + EV), vector + pBABE-miR-216a (EV + miR-216a), vector + pBABE-DANCR (EV + DANCR), or pBABE-DANCR + pBABE-miR-216a (DANCR + miR-216a) (*n* = 4, *t* test, * *p* < 0.05).

After verifying the competitive binding of DANCR and miR-216a, we hypothesized that DANCR could be regulating Wnt/β-catenin via miR-216a repression. To test this, we overexpressed DANCR by transfecting A549 cells with a DANCR cDNA vector. An increase in DANCR expression was detected 48 h after transfection (Figure 6A), which increased β-catenin protein expression compared to that in vector control cells (Figure 6B). After the first round of DANCR transfection, cells were expanded and transfected again with a vector control or miR-216a plasmid. We confirmed the overexpression of DANCR and miR-216a (Figure 6C). Western blot analysis revealed DANCR and vector-transfected cells increased β-catenin protein expression compared to vector and vector control cells, and the combination of DANCR and miR-216a decreased β-catenin expression. We consistently observed a decrease in β-catenin expression with the coexpression of DANCR and miR-216 compared to DANCR alone, although the decrease did not reach statistical significance (*p* = 0.06), lending support to our hypothesis that miR-216a regulates β-catenin (Figure 6D). These results suggested an interplay among DANCR, miR-216a, and β-catenin, and indicated that DANCR activates Wnt/β-catenin signaling through miR-216a repression.

## 4. Discussion

The Wnt pathway regulates key cellular functions such as proliferation and differentiation, and deregulation of its signaling can have widespread consequences [14]. Aberrant Wnt signaling has been observed in many cancer types, including lung cancer, and the overexpression or activation of Wnt is associated with poor prognosis. NSCLC tumorigenesis and metastasis are frequently associated with the upregulation of Wnt-pathway-activating genes and the downregulation of negative regulators [14,20]. However, the mechanisms that regulate Wnt signaling in NSCLC are unclear.

This study is the first to demonstrate that lncRNA-DANCR regulates the Wnt/β-catenin pathway through miR-216a inhibition, an established lung cancer tumor suppressor. Although recent studies showed DANCR regulation of Wnt/β-catenin signaling in glioma [21] and osteoblasts [22], this is the first report to establish this interaction in lung cancer cells, and the interplay of DANCR, miR-216a, and Wnt/β-catenin signaling.

DANCR expression was significantly increased in a panel of NSCLC cell lines, with the exception of H1944. The H1944 cell line could have developed and progressed from different mutations than those of the other lines, and DANCR overexpression was thereby unnecessary for its survival and growth. Further analysis of hundreds of NSCLC samples compared to normal lung tissue from the TCGA dataset demonstrated that DANCR is highly upregulated in NSCLC.

DANCR KD resulted in typical Wnt/β-catenin-inactivation features, including impedance in cell migration, the inhibition of long-term clonogenic potential, and a reduction in stem-like characteristics (Figure 2). While others reported that a reduction in DANCR acutely slows cell proliferation, delays the cell cycle, and increases apoptosis, we did not observe the same effects of transient DANCR KD on cell growth [12,13,23,24]. Some reports used transient knockdown methods with only 1 siRNA sequence [12,13], and proliferation inhibition and apoptosis promotion were only changed by approximately 10% [13]. Although we had comparable, if not better, transient reduction in DANCR expression as that in other studies, we discovered that the initial proliferation was largely unaffected (Figure 2B). A more consistent reduction in proliferation was reported in A549 cells, with stable knockdown of DANCR via shRNA [23,24], which we also observed by Day 4 (Figure S1B).

The difference in the effect of DANCR KD on long-term growth between A549 and H1755 may be due to inherent differences between the two cell lines. H1755 cells were derived from a metastatic lesion of adenocarcinoma, while A549 cells originated from a primary adenocarcinoma tumor. Moreover, our experiments revealed a nearly twofold increase in DANCR expression in H1755 compared to that in A549 (Figure 1B). As our analysis of TCGA data (Figure 1A) and previous reports suggested, increased DANCR expression is associated with poorer prognosis, and may potentially be related to more aggressive cancer types [6,7,8,9,12,21,24,25]. Therefore, higher DANCR expression in H1755 could have played a critical role in the pathogenesis of the originating H1755 lesion. This may explain why inhibition of DANCR expression produced a more significant effect on long-term growth.

We further showed that DANCR KD can inactivate β-catenin activity and reduce β-catenin protein expression (Figure 4). Our studies demonstrated that DANCR regulation goes beyond β-catenin, and extends to downstream effectors c-Myc and Axin2 (Figure 4). In addition, while DANCR and miR-216a interaction was previously reported in HEK293, breast, and hepatocellular carcinoma cells [12,25,26], we are the first to confirm this interaction in NSCLC (Figure 4B). Importantly, we observed that DANCR overexpression in A549 cells induced β-catenin protein expression, which could subsequently be blocked with miR-216a overexpression (Figure 6). This rescue effect illustrates the novel DANCR/miR-216a/β-catenin signaling axis in NSCLC cells, and it is therefore possible that miR-216a overexpression could inhibit DANCR-induced proliferation and migration. Upregulation of miR-216a was shown to inhibit cell proliferation and migration in a SCLC cell line, and inhibit NSCLC cell growth, invasion, and metastasis [3,27]. Furthermore, the in silico analysis (TargetScan, miRanda-miRSVR) of potential miR-216a targets determined several Wnt signaling molecules, including coreceptors Frizzled 4 and 5, and cyclin-dependent kinase 14 (CDK14), which activates Wnt signaling during the G2/M phase of the cell cycle. Therefore, it is likely that miR-216a normally keeps Wnt signaling in check, but DANCR overexpression in NSCLC effectively blocks the tumor-suppressive function of miR-216a.

Given the diverse functions and potential binding partners of long noncoding RNAs, it is also possible that DANCR activates Wnt signaling independent of miR-216a. DANCR was reported to associate to the *CTNNB1* gene in hepatocellular carcinoma, thereby protecting it from inhibitory miRNAs [6]; however, we were unable to detect an association under our conditions (data not shown). It is still possible that DANCR modulates β-catenin through interaction at the protein level or through the repression of Wnt inhibitors. Future studies should focus on identifying potential binding partners of DANCR and miR-216a to fully illuminate the relationship between DANCR and Wnt.

DANCR KD also significantly downregulated Sox2 gene and protein expression. Sox2 is an essential transcription factor that maintains stem-cell self-renewal and pluripotency [16]. The gene expression of *SOX2* is highly expressed in NSCLC subtypes, and the inhibition of Sox2 downregulates Wnt1/2 and c-myc gene expression, induces cell apoptosis, and reduces metastatic potential in lung cancer [28,29]. Our results provide evidence that DANCR may modulate Sox2 expression, but the mechanism is unclear (Figure 3A,B). One potential mechanism could be β-catenin-driven, as it was shown to bind and regulate the transcriptional activity of Sox2 in a small subset of breast-cancer cells [30]. Another study in breast cancer found that Sox2 and β-catenin synergistically promoted the transcription of *CCND1*, resulting in enhanced proliferation and tumorigenesis [31]. Others studies, however, showed that Sox2 antagonizes β-catenin signaling to maintain self-renewal in osteoblasts [32], mesenchymal stem cells [33], and osteosarcomas [34]. Therefore, the functional relationship between β-catenin and Sox2 may be cell-type- and context-dependent. Alternatively, another possible mechanism may involve miR-216a regulation. In silico analysis (miRanda-miRSVR) revealed Sox2 as a potential binding partner of miR-216a. Thus, if miR-216a does target Sox2 in addition to Wnt signaling molecules, it could implicate the underlying role of miR-216a in CSC regulation.

CSCs make up a small subpopulation of cancer cells that possess features of self-renewal, differentiation, and tumor initiation [35]. Failure in cancer therapies have been widely attributed to the CSC population due to their intrinsic ability to resist chemotherapy through the promotion of drug-efflux pumps and the maintenance of a quiescent state. Because DANCR increases NSCLC stemness through the activation of CSC regulators Wnt and Sox2, DANCR could also promote a chemoresistant phenotype. Thus, DANCR could potentially serve as a novel therapeutic marker to predict patient response to chemotherapy. Evidence of the prognostic value of DANCR is exhibited by its correlation with poor prognosis in NSCLC, and the promotion of tumorigenesis and metastasis in multiple cancers [11,12,24,36]. Future studies should focus on characterizing the role of DANCR in CSCs to determine its impact on chemoresistance, tumor initiation, and metastasis in NSCLC.

## Supplementary Materials

The following are available online at https://www.mdpi.com/2218-273X/10/12/1646/s1. Figure S1. (A) qRT-PCR measuring fold change of DANCR expression in stable A549 DANCR KD compared to scramble control (*n* = 3, *t* test, **** *p* < 0.0001); (B) cell growth of stable A549 DANCR KD cells compared to scramble control (*n* = 3, 2-way ANOVA, * *p* < 0.05, ** *p* < 0.01, *** *p* < 0.001, **** *p* < 0.0001); (C) cell impedance assay of stable A549 DANCR KD cells compared to scramble control (representative of 3 independent experiments); (D) colony formation of stable A549 DANCR KD cells compared to scramble control (*n* = 3); (E) qRT-PCR of DANCR expression in A549 cells cotransfected with DANCR shRNA + TOPFlash luciferase vector compared to scramble + TOPFlash luciferase vector (*n* = 3, *t* test, * *p* < 0.05, ** *p* < 0.01). Figure S2. Representative images of effect of DANCR KD on clonogenic growth in (top) A549 and (bottom) H1755 cells compared to scramble control.
