# Supplementary material for: Long Noncoding RNA DANCR Activates Wnt/β-Catenin Signaling through MiR-216a Inhibition in Non-Small Cell Lung Cancer
Source: Biomolecules. 2020 Dec 8;10(12):1646. doi: 10.3390/biom10121646 (PMC7764320; doi:10.3390/biom10121646)
Supplement: Supplementary file 1 [file biomolecules-10-01646-s001.pdf]

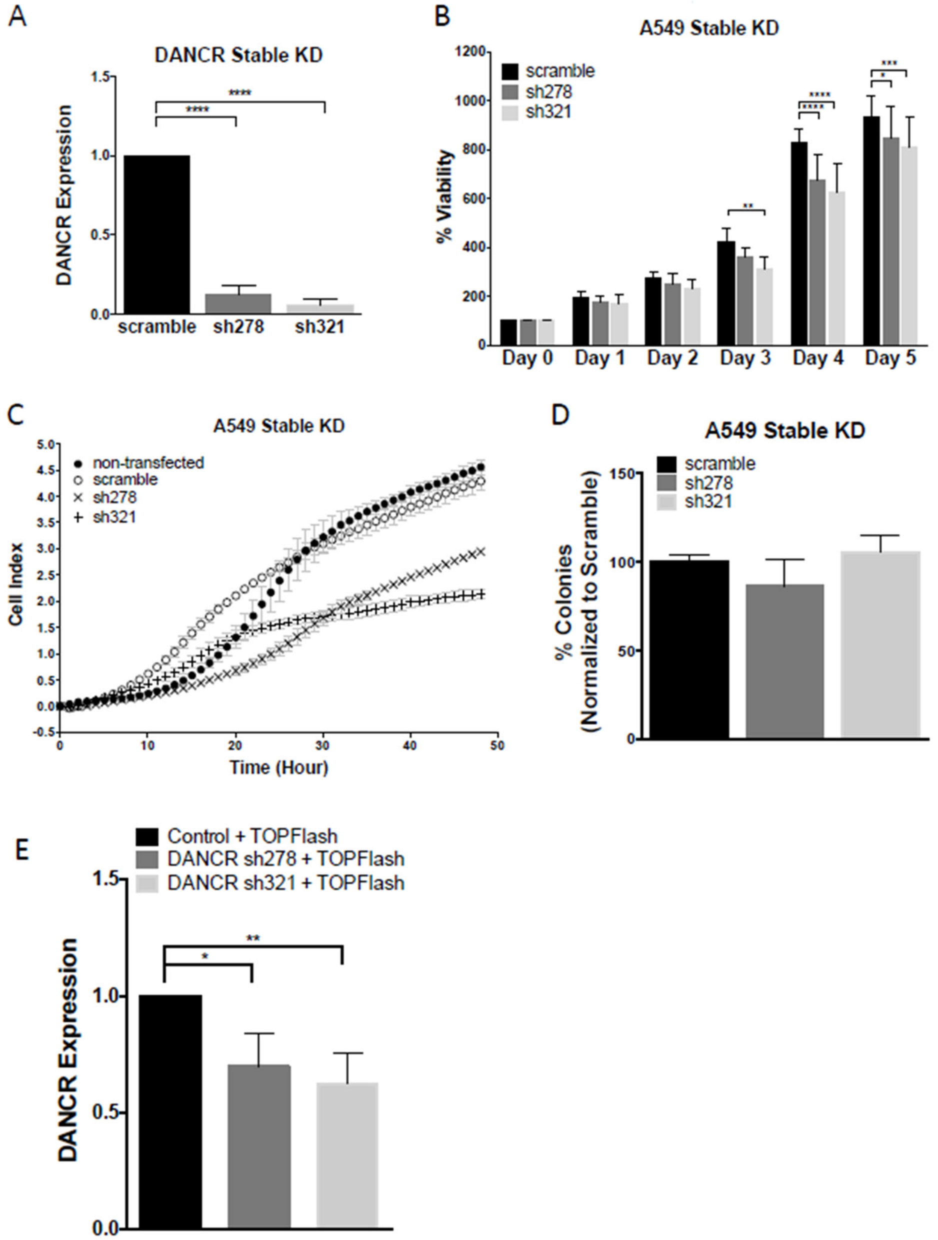

Figure S1. A) qRT-PCR measuring fold change of DANCR expression in stable A549 DANCR KD compared to scramble control (n=3, t-test, ****<0.0001); B) Cell growth of stable A549 DANCR KD cells compared to scramble control (n=3, 2-way ANOVA , *<0.05, **<0.01, ***<0.001, ****<0.0001); C) Cell impedance assay of stable A549 DANCR KD cells compared to scramble control (Representative of 3 independent experiments); D) Colony formation of stable A549 DANCR KD cells compared to scramble control (n=3); E) qRT-PCR of DANCR expression in A549 cells co-transfected with DANCR shRNA + TOPFlash Luciferase vector compared to scramble + TOPFlash Luciferase vector (n=3, t-test, *<0.05, **<0.01).

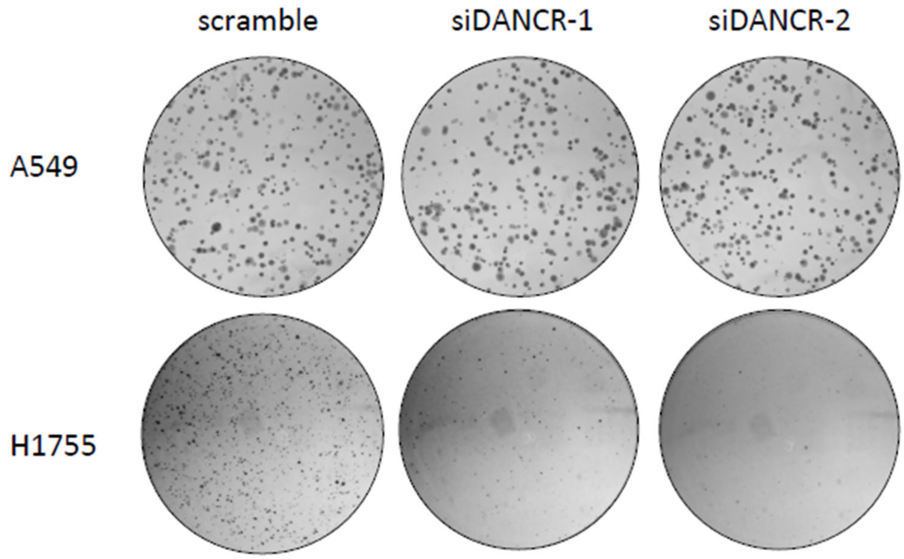

Figure S2. Representative images of the effect of DANCR KD on clonogenic growth in A549 (top) and H1755 cells (bottom) compared to scramble control.
